# Learning health system for implementation, scale-up, and sustainment: a systematic review to consolidate guidance for improvement

**DOI:** 10.1186/s13012-025-01482-z

**Published:** 2026-01-10

**Authors:** Cassandra Lane, Sam McCrabb, Heidi Turon, Caitlin Bialek, Lucy Couper, Magdalena Wilczynska, Samantha Gray, Courtney Barnes, Madeleine Fee, Tanja Kuchenmüller, Davi Mamblona Marques Romao, Luke Wolfenden

**Affiliations:** 1 School of Medicine and Public Health, The University of Newcastle, Newcastle, NSW Australia; 2 Hunter New England Population Health, Hunter New England Area Health Service, Newcastle, NSW Australia; 3 National Centre of Implementation Science, The University of Newcastle, Newcastle, NSW Australia; 4 Hunter Medical Research Institute, New Lambton Heights, NSW Australia; 5 Research for Health Science Division, World Health Organization, Geneva, Switzerland

**Keywords:** Learning Health System, Implementation, Scale-up, Sustainment, Evidence Synthesis, Framework Synthesis

## Abstract

**Background:**

Learning Health Systems (LHSs) link research and health service delivery by generating evidence to guide decision-making and continuous improvement. Although various LHS frameworks exist, there is limited practical guidance for how LHSs can improve implementation. This systematic review aimed to consolidate existing guidance to identify the infrastructure (pillars) and improvement processes (steps) required to support a LHS cycle that improves the implementation (including scale up or sustainment) of health programs, policies, or practices.

**Methods:**

We searched five databases and grey literature for documents describing an LHS model, or a process, or process model, guideline, or tool (i.e., guidance) intended to improve the quality of implementation, scale-up, and/or sustainment of health interventions. Title, abstract, and full-text screening were conducted independently by two reviewers. Data were synthesised separately for pillars and steps. Framework synthesis identified pillars and steps, informed by an existing LHS framework and refined iteratively; thematic synthesis explored patterns within each.

**Findings:**

From 12,151 records and 25 websites, 96 guidance documents were included. Six Pillars were identified as important to operationalise LHS improvement processes: 1-Interest holder engagement, 2-Workforce development and capacity, 3-Evidence surveillance and synthesis, 4-Data collection and management, 5-Governance and organisational processes, and 6-Cross-cutting infrastructure. The improvement process was comprised of 10 ‘Steps’ across three LHS phases: Phase 1) Knowledge to Practice -Identify and understand the problem; Decide and plan for action; Assess and build capacity; Pilot; Phase 2) Practice to Data—Execute the action; Collect data; Monitor and respond; Phase 3) Data to Knowledge- Analyse and evaluate; Disseminate; and Decide (continue, adapt, or cease improvement efforts). Despite the diversity in purpose and context across included documents, the consolidated steps and pillars were conceptually consistent, suggesting a shared foundation. Some contextual variation in emphasis and operationalisation was noted, particularly among guidance focused on scale-up or sustainment.

**Conclusions:**

This review consolidated LHS pillars and improvement steps to better implement, scale or sustain health interventions. Findings provide a structured yet adaptable approach for operationalising implementation-focused learning cycles within LHSs. It informs forthcoming WHO guidance, and supports more systematic, responsive use of evidence in health systems.

**Trial registration:**

The review protocol was prospectively registered on Open Science Framework (https://doi.org/10.17605/OSF.IO/V4JRC).

**Supplementary Information:**

The online version contains supplementary material available at 10.1186/s13012-025-01482-z.

Contribution to the literature

- Consolidates diverse learning health system (LHS) and implementation guidance into a practical implementation-focused LHS framework of six Pillars and ten Steps to support implementation, scale-up, and sustainment.
- Provides a structured yet adaptable model that can guide context-sensitive operationalisation of implementation-focused LHSs in varied health system settings.
- Moves beyond infrastructure descriptions by articulating the processes of improvement often missing from existing LHS frameworks.
- Positions LHSs as both users and producers of evidence, bridging implementation science and real-world system learning.

## Introduction

Health systems and communities could benefit substantially from interventions that effectively reduce major health burdens such as cancers and diseases [1, 2]. Yet these interventions are infrequently implemented, scaled up, or sustained in routine practice at the pace or consistency required to achieve their potential impact [3–5]. Persistent barriers include slow and fragmented processes of transferring evidence from researchers to end-users [6], and limited context-relevant, actionable guidance on how to best implement interventions in real-world settings [7]. Addressing these challenges require a fundamental shift in how evidence is produced and applied within health systems to enable timely responses to current and emerging health needs.

Recognising these issues, international reviews have called for better integration of the research and health system enterprises to strengthen health systems and improve research translation [8–10]. In Australia, for example, the landmark McKeon review [10] envisioned a health system in which research is *“…fully embedded in all aspects of healthcare to deliver: 'Better Health Through Research'”.* Such reviews recommend a reorientation of health systems to generate and apply evidence that improves quality and outcomes, supported by strategies such as targeted funding, workforce development, and investment in critical research infrastructure [8, 9].

LHSs provide a framework for which this integration can be achieved. First proposed by the US National Institute of Medicine in 2007 [11], LHSs are defined as systems where “…*science, informatics, incentives, and culture are aligned for continuous improvement and innovation, with best practices seamlessly embedded in the delivery process and new knowledge captured as an integral by-product of the delivery experience”* [12]*.* In an LHS, health services generate the evidence needed to address their own priorities, with new knowledge immediately available to inform decision-making to continuously improve health services and their impact.

Interest in LHSs has grown rapidly, with a marked increase in publications over the past decade [13], and significant investments by governments in infrastructure to reorient health systems around LHS principles [13–17]. In addition, numerous frameworks and conceptual models of LHSs have emerged which consistently emphasise the importance of foundational ‘pillars’ – such as supportive infrastructure, capabilities, culture and leadership—and continuous improvement cycles that leverage these pillars to drive data-informed healthcare [13–17]. Nonetheless, fully operational LHSs remain relatively rare, and their establishment continues to present a considerable challenge [18].

Many LHSs position implementation science as a core competency and actively embed implementation research within system activities [19–22]. Because health systems and services are ultimately responsible for the implementation of policies, programs, and practices, LHSs offer a promising approach to generating and applying contextually relevant evidence on how best to implement, scale, and sustain proven interventions. In doing so, LHSs advance the goals of implementation science—accelerating uptake of effective interventions, enhancing fidelity and reach, and reducing the time lag between discovery and routine care [7].

As such, implementation-focused LHSs—those that apply LHS principles to improve the implementation, scale-up, and sustainment of evidence-based health interventions (rather than focusing on discovery science such as genomic research, drug development, or analytics-only models) – hold considerable potential for strengthening health systems and improving patient and population health outcomes. While some examples exist [23, 24], there is limited guidance on how to operationalise LHSs specifically for implementation, scale-up, or sustainment. It is intended to be operationally focused, offering guidance to undertake actions to support (pillars), or undertake (steps) ongoing improvement processes to enhance implementation, (scale-up or sustainment) of health interventions, policies or programs. Calls have been made for more practical, operational guidance to support their development and functionality [18].

To address this gap, we conducted a systematic review and used qualitative evidence syntheses to produce an implemented-focused LHS framework. The synthesis consolidated existing LHS frameworks, and models and guidance documents that aim to generate and apply evidence to improve the implementation, and/or scale-up, and/or sustainment of health interventions (policy, practice, program). The resulting framework is intended to inform forthcoming World Health Organization (WHO) guidance on implementation focused LHSs and complements ongoing efforts by WHO and its Evidence-informed Policy Network (EVIPNet) to support Member States in strengthening the systematic and transparent use of evidence in policy [25]. It is intended to be operationally focused, offering guidance to undertake actions to support (pillars), or undertake (steps) ongoing improvement processes to enhance implementation, (scale-up or sustainment) of health interventions, policies or programs.

Our research aims were:

1. Pillars: to identify the infrastructure, expertise, and resources (pillars) recommended to support and enable implementation focused improvement processes – (e.g., ‘steps’); and to describe the core elements or functions within identified pillars.
2. Steps: to describe the steps or stages recommended to improve the implementation (and/or scale-up or sustainment) of health programs, policies, or practice; and to characterise the core actions or activities associated with each identified step.

## Methods

### Protocol and registration

This systematic review was conducted in accordance with best practice methods [26] and reported following the Preferred Reporting Items for Systematic Reviews and Meta-Analyses (PRISMA) 2020 guidelines [27]. The review protocol was prospectively registered on Open Science Framework (10.17605/OSF.IO/V4JRC). The protocol was developed by a team of Australia-based implementation and behavioural science researchers, with input of the WHO and a WHO convened Editorial Board of international experts established to support the development of evidence-based guidance.

### Eligibility criteria

Table 1 details the review eligibility criteria.

**Table 1:** Eligibility criteria

Criterion	Definition (eligible)
Types of documents	• Full-length peer reviewed publication or grey literature • Describing a process model, guideline, or tool for improving the quality of implementation, scale-up, and/or sustainment of health interventions or LHS
Guidance	• Deliberate description of a process to improve the impact of a health service, policy, practice, or program (intervention) • Deliberate description of a process or approach to implement an LHS • Deliberate description of how to achieve ongoing incremental improvements in the implementation of interventions, such as quality improvement processes • Relevant to a health or public health service, policy, practice, or program
Settings	• Applicable at a national, subnational/regional or facility level
Health condition	• Non-condition specific
Language	• No restrictions
Date	• No restrictions

#### Types of documents

We included documents published in the peer reviewed or grey literature that described an LHS model, or a process, or process model, guideline, or tool (i.e., guidance) intended to improve the quality of implementation, scale-up, and/or sustainment of health interventions. Guidance was defined as a deliberate description of a process to enhance the impact of a health service, policy, practice, or program – typically presented in a series of steps or stages that may or may not be linear [28, 29]. Full length documents – defined as those containing substantive narrative content [30]– were eligible for inclusion. Abstracts, conference proceedings, and letters were excluded. There were no language restrictions; articles published in languages other than English, French, Portuguese, Polish, or Spanish (spoken by members of the research team) were translated using Google Translate.

#### Types of guidance

Guidance documents were included if they intended to: i) improve the extent, quality, scale or completeness of the implementation of an intervention or LHS, or ii) sustain such improvements. This included guidance to achieve ongoing incremental improvements in the implementation of interventions (e.g., quality improvement processes) and guidance aimed at supporting implementation at scale. Guidance must have been relevant to a health or public health service, policy, practice, or program. Documents were excluded if they (i) focused solely on a specific condition (e.g., diabetes management plans) as these may not be applicable more broadly to health or public health policy, practice or program implementation, (ii) were developed for use outside of health or public health systems (e.g., in manufacturing or agriculture) as these types of guidance would not be specific enough to a health setting, or (iii) described implementation support activities without a structured process (e.g., training or communication of research findings) as we were aiming to synthesise structured processes.

### Search methods for identification of studies

We searched for publications from the last 10 years, although older studies were included if eligible and identified through backward citation searching (described below).

#### Electronic searches

We searched the academic databases MEDLINE (Ovid), PsycINFO (Ovid), and EMBASE (Ovid) in April 2024. Search terms were informed by terminology used in Doherty et al. for implementation research [31] and by terms related to LHSs [32] from the Canadian Health Libraries Association identified on the InterTASC Information Specialists' Sub-Group Search Filter Resource [33]. The search strategy was reviewed and edited by a Senior Research Librarian to ensure accuracy prior to database searching. A research design filter was applied to exclude citations of research trials [34]. The full search strategy is detailed in Supplementary File 1. Eligible documents were also drawn from a recent systematic review of adaptation frameworks [30], as well as any adaptation frameworks published since that review.

#### Searching other resources

In consultation with field experts, we developed a list of key websites to search, including departments within the WHO and other relevant agencies (Supplementary File 2).

#### Backwards citation searching

If an eligible document cited a more complete version of the guidance, efforts were made to locate and include.

### Data collection and analysis

#### Selection of studies

After removing duplicates using Endnote and Covidence, title and abstract screening was undertaken independently by two reviewers (SMc, HT, LC, MW, CBi, SG, CBa, KB, MD, BM, RH, NN) using Covidence systematic review software [35]. Full-text articles were retrieved for all publications deemed potentially eligible. Two members of the research team independently assessed the full texts for inclusion and extracted information from the eligible studies (SMc, HT, LC, MW, LW, CBa, KB, MD). Disagreements regarding eligibility were resolved through discussion and, when necessary, consultation with a third reviewer.

#### Data extraction and management

Data from all included studies were extracted by one review author and checked for accuracy by a second reviewer (SMc, HT, LC, MD, MF, MW, SG, KB, CBa). Any discrepancies were resolved through consensus or consultation with a third reviewer (SMc, HT, LC, MD, MF, MW, SG, KB, CBa). A bespoke data extraction form was developed and piloted by two reviewers to ensure consistency and usability. Data were extracted into two broad categories:

- Characteristics of guidance:
  o Citation information including name, details, year published.
  o Key attributes including intended World Income Level of the country the guidance originated in, stated purpose, intended audience, the discipline in which it originated, and at what level implementation is occurring.
- Details of the steps/phases including the number of steps, the listed steps and definitions, whether the process included a defined endpoint or was continuous, and whether the process was linear or non-linear.

#### Data synthesis

Synthesis methods were informed by the RETREAT criteria for selecting a qualitative evidence synthesis approach [36] and aligned with the aims of this review. NVivo software (v14) [37] was used for data management, coding, syntheses, and secondary analyses. Separate synthesis processes were undertaken for the Pillars and Steps objectives by two coding teams, under the direction of an experienced qualitative researcher (CL). To provide an LHS foundation, the codebooks for syntheses were structured based on the LHS Framework by Wolfenden et al [24] – which was developed based on a review of LHS frameworks by Menear et al [15] and recommendations for LHSs focused on implementation.

##### Pillars

Framework synthesis [38–40] was used to identify the infrastructure, expertise, and resources (pillars) recommended to support and enable implementation focussed improvement processes. The foundation of Framework Synthesis is a strong starting codebook to guide the coding process. The six pillar domains from Wolfenden et al.’s LHS framework [22] provided the preliminary codebook. All identified guidance documents were coded, starting with the preliminary codebook which was then iteratively developed. A four-person coding team (MW, SG, CL, LW) extracted content including text, table extracts, and figures, though preference was given to narrative content when content was duplicated across formats. Ten frameworks were initially double-coded to refine the codebook and support inter-coder consistency. Remaining frameworks were single-coded and independently checked by a second coder. Discrepancies were resolved through discussion or escalated to a third coder if needed. The coding team met regularly to review and iteratively refine the codebook. A final review and data cleaning were conducted by the synthesis lead (CL).

Thematic synthesis [41] was then used to describe the core elements or functions within each identified pillar. The synthesis lead (CL) analysed the coded content within each Pillar to inductively develop themes reflecting specific elements or functions. Original guidance document sources were revisited for clarification where needed, and final themes – presented as narrative summaries within each Pillar – were discussed and confirmed through team consensus.

##### Steps

Framework synthesis [38–40] was used to synthesise the steps or stages suggested by eligible guidance documents to improve the implementation of evidence-informed health programs, policies, or practice. The three phases of the LHS Framework by Wolfenden et al [24] (Phase 1) Knowledge to Practice; Phase 2) Practice to Data; and Phase 3) Data to Knowledge) formed the codebook. Because the phases are quite broad, additional structure was added to the starting codebook. One team member (SMc) first charted the steps described in the first 50 eligible guidance documents in Excel, mapping them against the LHS phases and identifying broad step categories. This initial mapping was checked by another team member (HT) and the output of this process formed the preliminary codebook. A team of four coders (SMc, HT, LC, CB) then followed a similar process to that used for Pillars synthesis: an initial ten guidance documents were double-coded, followed by single-coding with independent checks. The team met regularly to iteratively adapt the codebook, and the synthesis lead (CL) completed final checks and data cleaning.

Thematic Synthesis [41] was then used to characterise the core actions or activities described within each finalised step. As with Pillars, this was undertaken by the synthesis lead (CL) and involved inductive thematic coding of the content assigned to each finalised step to identify themes. Guidance documents were revisited where necessary for clarity, and the themes were finalised through team consensus. These discussions also prompted a deductive search for actions or activities that are commonly associated with each step but may not have emerged through the inductive process. This served as a quality assurance check to ensure a more complete and robust synthesis.

##### Secondary analyses (Coding comparison)

NVivo software was used to compare coding for Steps and Pillars based on the key attributes extracted (see "Data extraction and management" section).

##### Quantitative coding comparison

NVivo’s cross-tab query function was used to calculate the proportion of frameworks, grouped by key attributes, that contributed to each Step or Pillar. Three members of the team (CL, SMc, HT) visually inspected how much each subtype of a key attribute contributed to the coding of Steps and Pillars, exploring whether any subtypes disproportionately influenced certain Steps or Pillars. For example, whether frameworks from low and middle income countries were under- or over-represented in coding compared with high income countries. This served as a quality check for relative representation across types of frameworks.

##### Qualitative coding comparison

NVivo framework matrices were used to explore the qualitative content coded to each Step or Pillar by framework for the following attributes of interest: World Income Level of the country in which the guidance originated (low and middle income country versus high income country); Level of implementation (national or state policy, regulation, or law versus local or organisational policy, program, guideline); and purpose of the framework (implementation versus scale-up versus sustainability). This thematic analysis, by the synthesis lead, allowed identification of where steps or pillars may need to be operationalised differently according to different attributes. These analyses explored the question: does an LHS pillar or step have different functions or activities across countries of different income levels (high vs low and middle income); when undertaken at different levels of implementation (e.g., national versus local); or used for a different purpose (e.g., implementation versus sustainment)?

## Results

**Fig. 1:**
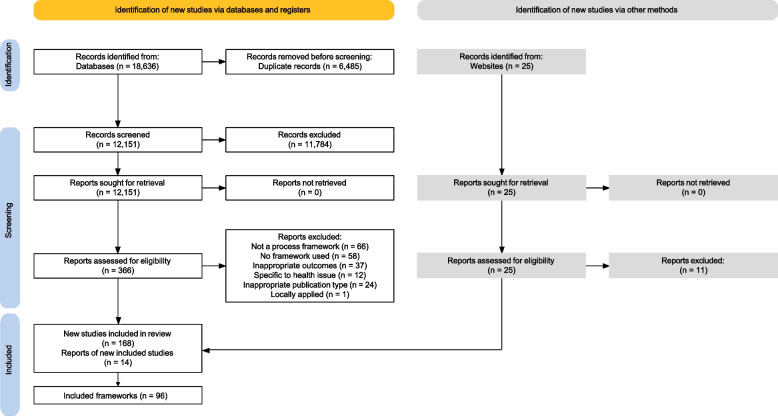
PRISMA flow diagram

Our search identified 12,151 unique documents and 25 websites. Following screening, 168 documents describing 96 guidance processes, models, or tools were included in this review [15, 24, 25, 42–134]. Supplementary file 3 provides details of each guidance document, including label, characteristics, and complete citation. Figure 1 displays the PRISMA flow diagram.

### Characteristics of included frameworks

Most included documents (*n* = 81; 84%) originated from high income country contexts, while a smaller number were applied in low- and middle-income country contexts (*n* = 7; 7%) or in both (*n* = 7; 7%). One (1%) did not include any information to determine the context in which it was applied. The majority were developed within the disciplines of health and public health (*n* = 66; 69%), and the primary purpose for most guidance was implementation (*n* = 68; 71%). Intended end users were predominantly clinicians (*n* = 45; 47%) or multidisciplinary professionals (*n* = 32; 33%). The number of steps outlined in each framework ranged from three to 24, with a mode of five and a median of six steps. Most frameworks were designed for implementation at the organisational policy level (*n* = 63; 66%), while 22 (23%) were applied at both the national/state and local/organisational policy levels and the remaining 11 (11%) at the national/state level. Summary characteristics of guidance documents are detailed in Table 2.

**Table 2:** Summary characteristics of included guidance documents

Characteristic	Details
Year published	Range: 1947–2024 Mode: 2024
Number of steps	Range: 2–24 Mode: 5
Applied in low- and middle-income country	Yes: 16 (17%) No: 80 (83%)
Country income level	High-income country: 81 (84%) Low- and middle-income country: 7 (7%) Both: 7 (7%) No information: 1 (1%)
Purpose of guidance	Implementation: 68 (71%) Scale-up: 7 (7%) Sustainment: 3 (3%) LHS: 11 (12%) Adaptation: (2%) Equity: (2%) De-adoption/implementation: (2%) Multiples: (2%)
Intended audience	Clinicians: 45 (47%) Multiple: 32 (33%) Governments/policymakers: 6 (6%) Public health practitioners: 5 (5%) Academics: 3 (3%) Businesspeople: 3 (3%) School teachers: 1 (1%) Implementation support practitioners: 1 (1%)
Discipline of origin	Learning Health Systems: 7 (7%) Implementation Science: 15 (16%) Health & Public Health: 66 (69%) Business/Engineering: 6 (6%) Unclear: 2 (2%)
Level of implementation	National or State policy, regulation, law: 11 (11%) Local or organisational policy, program, guideline: 63 (66%) Both: 22 (23%)

### Aim 1: Pillars

**Fig. 2:**
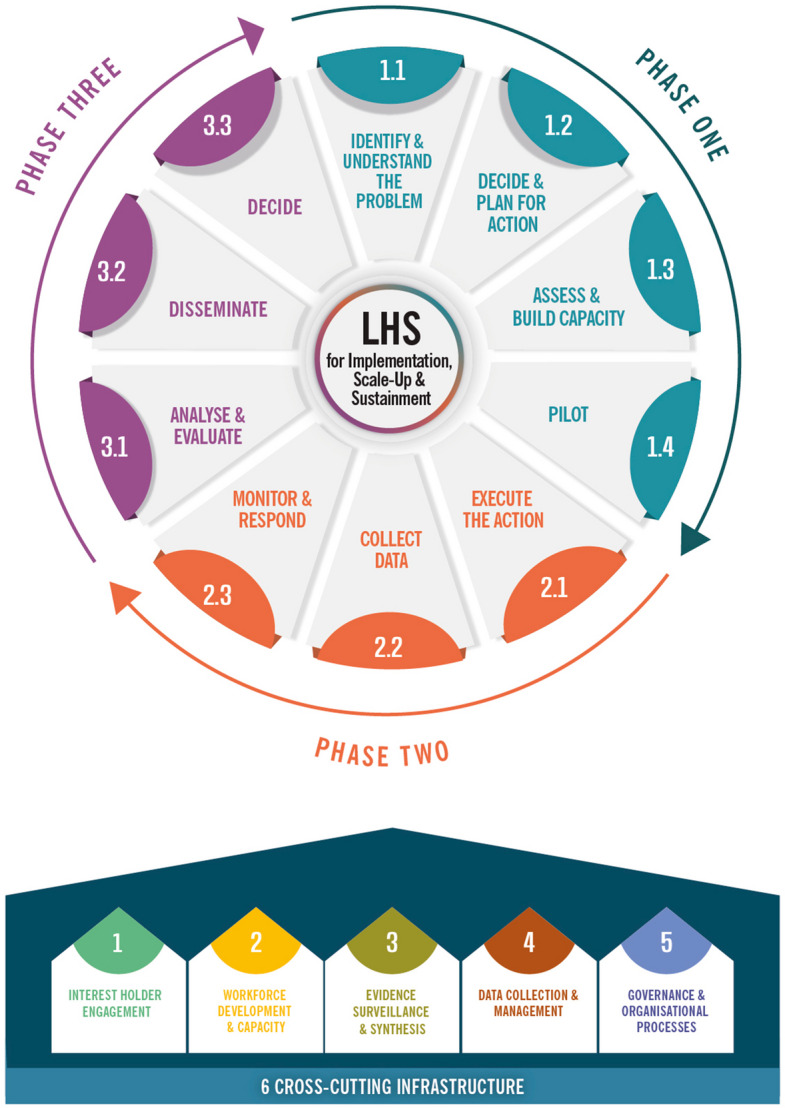
Learning Health System for implementation, scale-up, and sustainment

Figure 2 displays the resulting LHS for implementation, scale-up, and sustainment. Six pillars were identified to support and enable improvement processes. Coding presence for each Pillar across all guidance documents can be found in Supplementary File 4.

- Pillar 1 (Interest holder engagement)—strategies and supports used involve a diversity of people and organisations with the knowledge and skills, expertise and experience needed for LHS activities. This encompasses consultation, collaboration and partnerships.
- Pillar 2 (Workforce development and capacity) -systems and structures to build, strengthen, and sustain the capacity and capability of personnel involved in operationalising LHSs, including activities related to its pillars and improvement processes (cycles). This might involve mentoring schemes, training mechanisms, and human resource staffing initiatives.
- Pillar 3 (Evidence surveillance and synthesis)—systems, structures, and processes used to identify, monitor, and integrate relevant evidence generated outside of the LHS, with that generated within it to inform decision-making. This includes infrastructure needed to search, manage, and synthesise different forms of research from multiple and varied sources.
- Pillar 4 (Data collection and management)—systems and methods used to capture, process, and apply primary data, and includes (routine) data collection, management and analysis infrastructure.
- Pillar 5 (Governance and organisational processes)—structures, standards, culture, and leadership needed for the effective stewardship, operationalisation and coordination of LHS activities. This includes formal reporting, accountability and data governance processes, and regulations and standards to ensure safe and ethical conduct and manage risk.
- Pillar 6 (Cross-cutting infrastructure) – supports spanning multiple Pillars, encompassing Communication systems (information flow into, within, and out of an organisation); People, expertise and learning (the people involved in operationalisation); Funding and resources (financial, material, and structural supports); and Tools (specific instruments or resources).

Table 3 provides an overview of each Pillar and details of its core elements.

**Table 3:** Pillars used to support a Learning Health System improvement cycle (steps)

**1. INTEREST HOLDER ENGAGEMENT** Strategies to solicit the involvement of diverse people (see Pillar 6) with the necessary knowledge, skills, and experience needed to support the LHS activities. In particular, this supports decision making and serves as a bridge between research and practice
**Consultation** A structured process of gaining insight from interest holders, experts, consumers, or end-users to inform step activities. This may involve feedback mechanisms, expert reviews, facilitated discussions, and iterative engagement. Experts are often consulted at key points in the framework where technical expertise is needed
**Collaboration and partnerships** The strategic bringing together of diverse interest holders (e.g., policy-makers, researchers, practitioners, technical experts, and equity-relevant groups) necessary to support the framework. Engagement can be formal or informal, involving collaboration (co-creation, co-design, co-production) and coordination across all or specific LHS steps. Emphasis is placed on reciprocity and community-level insight to ensure relevance and LHS success
**2. WORKFORCE DEVELOPMENT AND CAPACITY** The systems and structures necessary to build, strengthen, and sustain the capacity and capability of personnel involved in operationalising the framework. This includes mentoring structures, training mechanisms, and HR staffing initiatives
**Mentoring structures** Structures designed to support the mentorship of personnel, typically involving a leader or someone in a position of authority, who guides, coaches, and/or leads by example to influence practices and behaviours
**Training mechanisms** Systems and processes designed to build the capacity of personnel, encompassing: (i) education to enhance understanding and motivation, and (ii) training to develop specific skills needed to operationalise one or more steps. These may be part of ongoing professional development or tailored education programs, commonly delivered via workshops, formal courses, expert facilitators, or train-the-trainer approaches. Various personnel may be targeted (e.g., front-line staff, data handlers, end-users) with an emphasis on the teams required to operationalise the steps
**HR staffing initiatives** Initiatives related to the recruitment and management of staff. This includes recruitment (e.g., position descriptions and selection criteria to ensure the necessary skills), contracting for expertise, and designating specific positions. It also encompasses ongoing staff management (e.g., supervision, work environments) and strategies for recognition and reward to retain and motivate personnel
**3. EVIDENCE SURVEILLANCE AND SYNTHESIS** The systems, structures, and processes used to identify, monitor, and integrate relevant evidence to inform decision-making. This includes surveillance mechanisms for tracking emerging data and synthesis methods for evaluating and consolidating information from multiple sources
**Evidence surveillance infrastructure** The infrastructure used to continuously identify, track, and monitor relevant secondary data and research that is available. This includes searches for systematic reviews, policy reports, national guidelines, and grey literature, often queried through online databases and platforms (e.g., MEDLINE, Cochrane, PubMed)
**Evidence synthesis infrastructure** The infrastructure used to synthesise secondary evidence, integrating data from various sources, including scientific literature, policy reports, and grey literature, to inform decision making. Tools and methods such as systematic reviews, meta-analyses, and qualitative synthesis are often employed, along with supports that facilitate this collation (e.g., collaboration with research experts, librarians, automated screening tools)
**4. DATA COLLECTION AND MANAGEMENT** The systems and methods used to access, capture, process, and use primary data
**Data collection infrastructure** The infrastructure used to gather data as the primary evidence source for a given step. This may involve existing data sources (e.g., health records, national surveys) or newly established data sources (e.g., needs assessments, qualitative inquiries). Infrastructure is required to ensure data for a broad range of outcomes can be collected (e.g., measures of effect, process measures, cost etc.) Data collection infrastructure may include those needed for the conduct of experimental studies (e.g., clinical trial infrastructure to support randomised controlled trials implementation trials) as well as routine tracking and interest holder-reported data (e.g., participant surveys), with an emphasis on both qualitative and quantitative approaches. Ideally, infrastructure allows data collection to occur in a timely, ongoing, valid and reliable way, at minimal cost. Established methods for routinely collected data (such as electronic health records) are commonly used in many LHS
**Data management and analysis infrastructure** The infrastructure used for secure storage and analysis (e.g., statistical methods, comparisons, or evaluations) to generate insights and support decision making. This includes hardware, software or other platforms to support linkage of data across different data collection platforms and sites; and informatic infrastructure to process and support interpretation of complex data inputs (e.g., Artificial Intelligence) It may also enable real-time feedback mechanisms and data quality monitoring to enhance usability and reliability of primary evidence
**5. GOVERNANCE AND ORGANISATIONAL PROCESSES** The structures, standards, culture, and leadership of an organisation that ensure the effective operationalisation and coordination of activities
**Organisational structures** The formal design and structure of an organisation, including governance, decision making and accountability, defined roles and responsibilities, and operational procedures to support the implementation of LHS activities. Structures should be efficient, transparent, adaptable to change, and inclusive of interest holders (Pillar 5)
**Culture and leadership** Culture—The shared values, goals, beliefs, and norms that shape daily practices, interactions, behaviours, and perceptions within an organisation. A culture conducive to the LHS supports a shared vision, fosters learning and knowledge sharing, promotes continuous improvement, and emphasises reciprocity, teamwork, and collaboration among staff. Culture is strongly influenced by leadership – those individuals or teams who support, enable, or actively drive the LHS, either as part of their existing role or through designated responsibility. Effective leadership is characterised by attributes such as authority, influence, interpersonal and communication skills, and the ability to motivate and inspire others
**Regulations and standards** Existing, modified, or newly developed regulations, policies, guidelines, and/or standards to support operationalisation of the LHS. These are applicable across various levels, including legislative, system, and organisational contexts. Examples include ethical guidelines and processes, patient privacy provisions and medico-legal protections, financial policies, terms of reference, and data governance and safety standards
**6. CROSS-CUTTING INFRASTRUCTURE** The infrastructure that extends across multiple Pillars, including Communication systems; People, expertise, and learning; Funding and resources; and Tools. While they may not be explicitly present in every Pillar, they have the potential to influence and enable functions across various Pillars
**Communication systems** The continuous flow of information into, within, and out of an organisation. Information intake involves gathering insights e.g., via research, listening to interest holders, and leveraging networks. Dissemination can occur external to the organisation (e.g., publications, reports, presentations, media, and social connections); and/or internally (e.g., dashboards, leadership, team dialogue, staff training, and marketing strategies) There is an emphasis on formal meetings with operational personnel, as well as multi-directional communication channels, such as feedback loops, to ensure that information is not only shared but also acted upon
**People, expertise and learning** The people, individuals and/or groups—collectively referred to in some instances as the "learning community"—involved in the operationalisation of the framework. These can be existing entities supported to take on LHS responsibilities or newly established for this purpose. People are strategically chosen to ensure the right expertise and insight are available, encompassing a broad range of roles, such as academics, researchers, frontline staff, funders, end-users (including clinicians, providers, patients, and consumers), champions, change agents, and administrative and technical support. Engagement extends to local and community levels to ensure contextual relevance of the framework. People may be formally united into a group for driving the framework, such as multidisciplinary teams, taskforces, or advisory committees
**Funding and resources** The financial, material, and structural supports required to operationalise the framework. Funding includes internal and/or external investments, such as research grants and local funding streams, and in-kind support. Resources encompass essential elements such as adequate time, technologies, physical space, and technical resources required for specialised activities. Where appropriate, incentives, both monetary and nonmonetary (e.g., recognition and reward), may also play a role in the engagement of people (as above)
**Tools** Specific instruments or resources used to operationalise a particular step or function within the LHS. These may include project plans, decision aids, or data-related tools, as well as frameworks (e.g., theoretical frameworks) or guidelines (e.g., best practice guidance) that guide activities. Tools can be pre-existing or developed specifically for the LHS

### Aim 2: Steps

Ten Steps were identified across a three-phase LHS improvement cycle. Table 4 provides an overview of each Step and details of its core actions and activities. Coding presence across guidance documents can be found in Supplementary File 5.

**Table 4:** Steps used across a three-phase Learning Health System improvement cycle

**PHASE I—KNOWLEDGE TO PRACTICE**
**1.1 IDENTIFY AND UNDERSTAND THE PROBLEM**
**Identify the problem** The process begins by identifying the problem/s or areas requiring improvement. This can be approached in two ways: • Deductively, if an implementation, scale-up, or sustainment ‘problem’ has already been identified, gather evidence to confirm the issue and establish a baseline • Inductively, if a problem has not yet been brought to attention, gather evidence to identify an opportunity for improvement Evidence for problem identification or confirmation can be acquired through various approaches: • Engage with key interest holders (e.g., staff, end-users, and decision-makers) through discussion, consultation, or formal research. This can help to identify issues and to guide the problem identification process (e.g., providing insight on potential data sources) (Pillar 5) • Collect and analyse relevant data from existing sources, such as medical records, performance metrics, or other routinely collected data. If available data is insufficient, conduct targeted data collection to fill in gaps (Pillar 1) • Review the broader evidence-base, including existing research, standards, guidelines, and current practices (Pillar 4). This helps identify specific gaps or problems and assess whether recommended practices are being followed or if areas require improvement **Understand the problem** Clearly define the problem in terms of what it is (the issue to address), where it occurs (the context), and who is involved (the key decision makers, interest holders, or target groups). If >1 problem has been identified, prioritisation may be required. Gather information to understand the context and conditions surrounding the identified problem (Pillar 1). This provides the foundation for subsequent steps. Key factors to understand may include: • Contextual factors that could influence improvement efforts; • Organisational strengths and weaknesses that can drive or hinder improvement efforts; and • Knowledge/perspectives/behaviours of interest holders regarding the problem (Pillar 5) Formal situational analysis approaches such as needs assessments and Strength, Weakness, Opportunity, Threat (SWOT) analyses can be used to gain a comprehensive understanding of key contextual factors. Qualitative insights are especially valuable in capturing real-world experiences and providing a deeper understanding from interest holders (Pillar 5). Supplement local data by consulting experts and exploring the broader evidence base (e.g., peer-reviewed literature, grey literature) to identify barriers, facilitators, and successful strategies used in similar contexts (Pillar 5)
**1.2 DECIDE AND PLAN FOR ACTION**
**Define the scope** Establish the aims and objectives of the improvement effort, clearly defining what the project intends to accomplish. Detail the operational parameters for the improvement effort. Doing so can help focus resources on a feasible area of impact, ensuring the project remains realistic and achievable within the available resources and time frame **Develop an improvement strategy** Determine the most appropriate course of action to achieve the aims and objectives. This requires the development of an improvement strategy and a plan to action it. The improvement strategy should be evidence-based, feasible, practical, and appropriate within the context identified in Step 1.1. Developing an improvement strategy involves: i. Involvement of relevant interest holders in the strategy development and decision-making process, for example, through co-design processes (Pillar 5); ii. Using local or other data and research evidence to identify factors (e.g., barriers) that may impede achievement of the targeted outcomes of the improvement effort, that is, enhanced implementation, scale-up, and improvement (Pillars 1 and 5); iii. Reviewing the evidence-base for effective strategies that may overcome identified barriers. This should focus on peer reviewed research (e.g., systematic reviews) and best practice guidelines, but may also incorporate the findings of other relevant local evaluations (Pillar 4) iv. Using information gathered, and appropriate theory-based frameworks, to develop an improvement strategy to enhance implementation, scale-up, or sustainment of the intervention. The development process may involve the adaptation and/or tailoring of the intervention (or implementation, scale-up, or sustainment strategies) to ensure it is feasible, practical, acceptable, and appropriate for the context identified in Step 1.1. This should be documented in an improvement logic model **Plan for action** Develop a detailed plan to operationalise the improvement strategy and its evaluation. It should include key actions, timelines, necessary infrastructure, governance and organisational processes, including clear articulation of roles and responsibilities of involved parties; leadership and accountability mechanisms (Pillar 3); resources (e.g., funding, expertise); and communication processes (Pillar 6) The plan should describe specific details related to the delivery or implementation of the improvement strategy (and intervention), and a protocol for related evaluation activities in Phase II and III, such as: the outcome measures used to assess whether the goals and objectives of the improvement strategies have been achieved; how this data will be collected, managed, and analysed (in Step 2.3); and how the findings will be evaluated, inform decision making (Step 3.2), and disseminated (Step 3.3) **Identify a team** Identify a team to drive improvement efforts—an operational group or designated personnel responsible for executing improvement activities and their evaluation. This team may be overseen or guided by an existing organisational structure, or a project specific steering or advisory committee, providing strategic direction, expertise and mechanism of accountability. Key approaches to establishing a team could include: • The use of an existing team for this purpose (e.g., existing quality improvement team); • Formation of a subgroup within an existing organisation entity or structure; or • Establish an entirely new team, which may require recruiting new personnel and/or realigning or redistributing responsibilities among existing staff, and establishing roles, responsibilities, reporting and accountability mechanisms Team membership should be strategically aligned with the objectives and scope of the improvement effort, with consideration given to: the required expertise (often multidisciplinary); member credibility and influence; relevance of experience; contextual knowledge, and; commitment to the improvement objectives (Pillar 6). Leadership is a critical element of successful improvement efforts and extends beyond competent stewardship of the improvement team, to developing a supportive team culture, and modelling an explicit commitment to the improvement effort, values, objectives and outcomes (Pillar 3). Strategies to identify and constitute an effective team include: • Assess existing teams – evaluate whether a currently existing team has the necessary characteristics and responsibilities. Determine if additional roles need to be filled (Pillar 6) • New hires – recruit team members with the specific skills, experience, credibility, perspectives or knowledge required for the improvement effort (Pillar 2) • Training and education – provide training or development opportunities for current staff to equip them with the necessary skills required by a team (Step 1.3) (Pillar 2) • Consulting or contracting – bring in external expertise through consultants or contractors when specialised skills are required (Pillar 6) • Engage interest holders – as below (Pillar 5) **Engage interest holders** Identify key interest holders (Pillar 5 and Pillar 6) who hold influence over systems and processes that may determine the success of the improvement effort. Interest holders provide valuable insights; can provide team access to requisite skills or expertise; and mobilise resources to support the improvement process. This often begins with identifying the relevant groups or individuals who ‘hold an interest’ in the effort. This can be undertaken using formal processes such as interest holder analyses or actor mapping. Some interest holders may have been consulted in earlier Steps, contributing to problem identification and/or context assessment (Step 1.1). Broadly, key interest holders to consider include: • Those with the ability to influence decisions (e.g., policy-makers) • Those from the population, services, setting or context where the need for change is identified, encompassing: o Those who are responsible for implementing or delivering health services (e.g., clinicians, practitioners, health service providers) o Those who receive the intervention, experience intended outcomes or otherwise affected by the initiatives (e.g., end-users, consumers, patients) o Leaders, change agents, or champions who can facilitate change, influence peers, and advocate for the action/intervention/change o Priority populations (e.g., underserved or historically marginalised groups) At a minimum, establishing connections (Pillar 5) and communication systems (Pillar 6) with interest holders is essential. Building respectful, trusting and more meaningful partnerships early is recommended. Formal engagement can take various forms, including: • Inclusion in the operational team, • Participation in advisory groups or steering committees, and/or • Involvement in co-designing strategies (as above), piloting new initiatives (Step 1.4), or providing ongoing feedback during the process (Step 2.2)
**1.3 ASSESS AND BUILD CAPACITY**
Ensure that the foundational elements and pre-conditions for successful execution of the action plan are in place **Assess capacity and identify gaps** First, assess capacity and capability, for improvement among key individuals, organisations, or entities engaged in the execution of the improvement strategies. This includes assessment of individual factors (e.g., knowledge), organisational factors (e.g., ‘organisational readiness’), broader system characteristics, and the accessibility and availability of the required infrastructure to operationalise the improvement strategy. Environmental scans and other processes can be useful to help identify organisations, infrastructure or resources that could support improvement efforts. Identify capacity or capability gaps found through the process **Build capacity** Actively work to address identified gaps. This could occur through targeted professional development or training, strategic recruitment, contracting or commissioning specific expertise or infrastructure, forming new partnerships or other means Key considerations include: • Human capacity and capability (Pillar 2) – Teams and personnel should have the skills, knowledge, motivation, and buy-in to support implementation. Building human capacity and capability may involve recruiting staff with relevant expertise, engaging external experts for guidance, or providing training and professional development opportunities • Supportive culture and leadership (Pillar 3) – A workplace environment that fosters engagement, continuous learning, use of data, accountability, and sustained effort. Building supportive culture and leadership may be facilitated through organisational transparency, good governance and communication, securing leadership support and advocacy for improvement across levels of a system, aligning organisational vision with improvement goals, and reinforcing the purpose and benefits of the effort • Material and structural supports (Pillar 3) – Ensuring the necessary resources (e.g., technologies, space, materials) are in place. Building material and structural support may involve access to appropriate equipment, software, or digital tools, or strengthening and re-aligning systems and processes to support data collection and analysis activities
**1.4 PILOT** (*Optional*)
Ideally, seek to pilot or pre-test the improvement strategy and its processes before full execution in Phase 2 to identify areas for improvement and allow refinement to maximise its potential beneficial impacts Piloting would typically be undertaken on a smaller scale than planned when at full operation (e.g., within a single hospital or network) or might focus on pre-testing specific elements of the improvement strategy (e.g., materials, delivery methods) This may involve formal pilot research and evaluation or informal assessments. Involving the interest holders in this Step may foster early engagement, input and buy-in. Key indicators often examined during piloting include assessments of feasibility and acceptability (is the strategy suitable and well-received by those involved); its potential impacts (is the strategy showing early signs of achieving its intended outcomes/objectives); as well as processes and organisational factors (what are the *unidentified* operational, structural, and contextual factors impacting improvement that may need to be addressed). If the intervention or improvement strategy has been tailored (in Step 1.5) to enhance contextual fit, piloting can also confirm the appropriateness of such adaptations
**PHASE II – PRACTICE TO DATA**
**2.1 EXECUTE THE ACTION**
Execute the plan for action (improvement strategy) from Step 1.2. This will require the co-ordinated mobilisation of actions within the improvement team and of other interest holders or organisations engaged in strategy co-ordination or delivery. Too, the transition of focus of the improvement team, associated governance structures (Pillar 3) and communication processes (Pillar 6) from planning to an execution phase is required. While the focus of this step is on the execution of the improvement strategy with fidelity; flexibility and responsiveness to unanticipated challenges, or changes in context is required (Step 2.2)
**COLLECT DATA**
Collect data to enable evaluation of the improvement strategy (Pillar 1). Whether existing, modified, or newly developed, measures of key improvement outcomes should be sufficiently valid for their intended purpose Data collection should be purposeful, ensuring it is timely (near real time), conducted by appropriate personnel (Pillar 6), and comprehensive – capturing key outcome measures aligned with the goals and objectives identified in Phase 1 and to guide ongoing improvement. Both qualitative and quantitative data are important Key outcome measures for consideration include: i. Effect or impact (i.e., data to assess overall success of the strategy in improving implementation, scale-up or sustainment; or any adverse or unintended effects) ii. Process measures (i.e., data to understand how well the strategy was delivered (fidelity), who it reached and what contextual factors may have influenced this) iii. Determinants/Mechanisms of effect (i.e., data to identify how the strategy worked or was effective such as its impacts on addressing targeted individual, organisational or system barriers or outcome determinants) iv. Experiences and perspectives of individuals or groups involved (i.e. data to describe the experience of those affected by the strategy such as satisfaction or acceptability) v. Efficiency and costs of the strategy (i.e., data enabling economic evaluations to determine the absolute costs, cost effectiveness or efficiency of the strategy) vi. Other outcomes deemed pertinent (e.g., sustainability, differences between sub-groups)
**2.3 MONITOR AND RESPOND**
Even with thorough planning and preparation in Phase I, significant challenges are often encountered early in improvement efforts. Mechanisms should be in place to monitor and quickly identify such issues, appraise risks and prioritise a response (Pillar 3). Addressing such issues early can minimise disruption and prevent these from more systemically undermining improvement efforts. Systems to monitor for such issues could be either: • Formal—The establishment of formal data collection systems to assess and appraise key strategy implementation processes (or responses to them) (Pillar1) • Informal—Remaining attentive to insights or feedback from the team or interest holders regarding what is not working, allowing for quicker responses to emerging issues (Pillar 5 and Pillar 6)
**PHASE III – DATA TO KNOWLEDGE**
**3.1 ANALYSE AND EVALUATE**
Analyse Phase 2 data (Pillar 1). Comprehensive analyses support robust strategy evaluations and generates new insights for further improvement. Analyses should be pre-planned, and based on the improvement efforts aims and objectives (Step 1.5). The primary purpose of analysis of Phase 2 data is to assess the extent to which the improvement effort achieved its objectives in enhancing implementation, scale-up and/or sustainment of the targeted intervention. Insights gathered through analysis of process can support the interpretation of strategy effects, for example, by identifying if the strategy was indeed executed as intended, who it reached and how it was received Importantly, analyses of Phase 2 data can identify how the improvement strategy can be improved. For example, understanding the mechanisms of the improvement strategy (how it did or did not work) enables the introduction of future strategy elements that target factors found to be positively associated with effectiveness (determinants); and/or the discarding of strategy elements targeting factors that are not. Capturing the perspectives of those engaged or impacted by an improvement effort can aid adaptations that may strengthen strategy acceptability or satisfaction and increase ongoing engagement. Finally, analysis of costs data can help with decisions to optimise the impacts of improvement efforts given fixed or finite resources The effects of an improvement strategy are unlikely to be uniform across the health service or system where it is introduced, and so examination of differences in such effects across different patient, organisational, or system characteristics (e.g., subgroup analyses) is suggested to ensure improvement efforts do not generate or exacerbate inequities Some questions for consideration: i. Did it have the intended impact/succeed in its objectives? *(Effect or impact)* ii. Was the improvement strategy delivered as it was intended? Who did it reach? *(Process measures)* iii. What were the key determinants of the effects observed from the implementation strategy? *(Mechanisms of effect)* iv. What are the experiences and perspectives of those individuals or groups involved? How did they engage with the strategy? *(Experiences and perspectives)* v. What was the associated cost? *(Cost)* vi. Were the benefits observed equitable? *(Other outcomes)* This information is helpful to determine the value of efforts and provide information for next steps, including informing interest holders (Step 3.2) and determining how best to move forward (Step 3.3)
**3.2 DISSEMINATE**
Share findings and insights through appropriate communication channels (Pillar 6), considering the setting, goals, and objectives of the improvement efforts. The form of communication (source, content, mode) should be tailored to the targeted audience and be aligned with the communication purpose. This may include to: • Enhance implementation, scale-up or sustainment of the intervention by building awareness and knowledge of those involved in: i) intervention delivery (e.g., clinicians); ii) the execution of the implementation strategy (e.g., interest holders); or iii) others who can influence it (policy-makers and other decision makers) • Feedback findings internally with interest holders and through governance structures (Pillar 5) to ensure accountability; build capacity and strengthen relationships (targeting individuals and teams involved in the process) • Share knowledge broadly, to support and inform decision making of interest holders (including the public), or other health services and systems seeking to improve implementation, scale-up or sustainment of similar interventions. The findings should also be disseminated to researchers to contribute to the collective evidence-base
**3.3 DECIDE**
Reflect on the findings from Step 3.1 and any feedback from Step 3.2 and determine how best to move forward. Even following comprehensive analyses of program data, synthesising, and interpreting findings across different outcomes and data sources is complex. In many cases uncertainty may remain. A range of interest holders should be engaged in sense making and interpretation of evaluation findings The use of structured processes is suggested to ensure decisions are made using the appropriate program data and analysis (Pillar 1), with consideration of appropriate external evidence (Pillar 4), and with input from all relevant interest holders (Pillar 5) Broadly, decisions will need to be made regarding whether to: • Cease the improvement effort. Cessation of improvement effort may be due to: a) the improvement strategy having achieved desired outcomes in sufficiently enhancing implementation, scale-up or sustainment. In such instances, health services or systems may seek to monitor key outcomes (Pillar 1) to ensure such improvements do not erode over time. b) the improvement strategy not having adequately enhanced implementation, scale-up or sustainment, but further modification of the improvement strategy (e.g., via engaging in another improvement cycle) is considered unlikely to yield improvements of sufficient magnitude to warrant the investment; c) there was evidence of potential harmful or adverse effect or risk to patients, interest holders, health services or the health system • Continue the improvement efforts to further enhance beneficial impacts to patients, services or the health system. This could include transitioning the focus of the improvement effort, for example, from implementation to scale-up; or from improving the effectiveness of the strategy to enhancing its contextual fit, efficiency, acceptability, or reducing its costs. Continuation of improvement efforts will draw on opportunities identified for improvement following analysis of data in Step 3.1, and its interpretation. Ongoing improvement cycles will benefit from the existence of infrastructure, partnerships, evidence and other activities developed and undertaken prior, particularly those in Phase 1. As such, it may not be necessary to replicate all ‘steps’ for each following improvement cycle • Start a new cycle to implement, scale-up or sustain a different evidence-based intervention

Phase 1) Knowledge to Practice, comprised four steps.

- Step 1.1 (Identify and understand the problem)—uses evidence to identify problem/s requiring improvement, understanding its impacts and context.
- Step 1.2 (Decide and plan for action)—develops an evidence-based strategy to address the identified problem/s; creates a detailed plan to operationalise the strategy and its evaluation, and identifys a team and interest holders to drive improvement efforts.
- Step 1.3 (Assess and build capacity)—assesses capacity and capability to execute the action plan, and then actively works to address identified gaps – building capacity of the team, interest holders, and systems.
- Step 1.4 (Pilot) is an optional step pilot or pre-test of the strategy before full execution, allowing refinements.

Phase 2) Practice to Data, comprised three steps.

- Step 2.1 (Execute the action)—mobilises the team and infrastructure to execute the action/improvement strategy.
- Step 2.2 (Collect data) – gathers data to evaluate impacts.
- Step 2.3 (Monitor and respond)—monitors and responds to emerging challenges.

Phase 3) Data to Knowledge, comprised three steps.

- Step 3.1 (Analyse and evaluate)—analyses data to assess the extent to which the improvement effort achieved its objectives. This data is used to identify opportunities to further improve the strategy.
- Step 3.2 (Disseminate)—shares findings and insights internally or externally.
- Step 3.3 (Decide)—reflects on the findings and determines whether to cease, continue (by initiating another cycle), or shift the focus of the improvement effort.

### Secondary analyses

#### Quantitative coding comparison

Supplementary File 6 (Pillars) and 7 (Steps) display the results of the Crosstab Queries. As expected, slight variation in coding presence across Steps reflected the diverse nature and purpose of the guidance documents. For instance, some guidance focused heavily on early-phase planning (Phase 1; e.g., intervention design and preparation) while providing limited or no detail on subsequent phases. The highest coding presence was observed in Step 1.2 (Decide and plan for action; 99%), followed by Step 1.3 (Assess and build capacity; 58%). The lowest presence was for Step 2.3 (Monitor and respond; 14%) which was more commonly observed in general recommendations or embedded within other steps, indicating perceived importance, rather than explicitly labelled as a step or recommendation.

When examined by framework characteristic, Step 1.1 (Identify and understand the problem) showed higher coding presence in local or organisational guidance (62%) compared to national or state-level guidance (27%). In other areas, meaningful comparisons were limited due to small sample sizes; for example, only seven guidance documents originated from low- and middle-income countries, and only three were intended primarily for academic audiences. Some coding absences were also conceptually appropriate; for example, frameworks focused on scale-up or sustainability often did not include Step 1.1, as these phases typically assume that the problem has already been identified.

For the Pillars, Pillar 6 (Cross-cutting infrastructure) had the highest coding presence, particularly for Communication systems (76%) and People and expertise (78%). Other highly represented Pillars included Pillar 5 (Governance and organisational processes; 83%) and Pillar 1 (Interest holder engagement; 70%). One observed difference was that Pillar 3 (Evidence surveillance and synthesis) was more commonly coded in national, or state-level guidance documents compared to local or organisational ones (82% vs 43%). Again, comparisons by framework characteristics were limited by small denominators.

Overall, the proportion of coded content for each Step and Pillar was relatively consistent across guidance documents with different characteristics. While some variability was observed, there were no notable outliers or dominant patterns. This suggests a balanced contribution from the diverse range of frameworks included in the review. This lends confidence that the final synthesis was not disproportionately influenced by any framework subtype and reflects a broad and inclusive representation of perspectives.

#### Qualitative coding comparison

Final Steps and Pillars were consistently applicable across all guidance subtypes, indicating strong coherence and relevance despite the diverse guidance documents. This supports a shared conceptual foundation for LHS improvement processes. While the overall structure of the Steps and Pillars was broadly transferable, the analysis also revealed nuanced differences in how they were applied or emphasised within certain subtypes of guidance. These distinctions were not observed between guidance documents by income setting (low- and middle-income country vs. high income country). Instead, distinctions were more evident across guidance subtypes, particularly based on the intended purpose of the guidance (e.g., scale-up or sustainability) or its policy orientation (e.g., national or state policy versus local or organisational policy).

For example, while the content of the Steps and Pillars was largely consistent across national or state policy and local or organisational policy guidance, a notable difference emerged in the sequencing of activities. In some local/organisational policy guidance documents, the process began with the identification or establishment of an operational team (e.g., Adapted Intervention Mapping [AIM] in schools [135]) to support implementation strategy selection and use in healthcare settings [136], which then drove subsequent planning and implementation activities. This reflects one of the more challenging steps to place in a standardised sequence and is discussed further in the main discussion section.

Guidance documents focused specifically on scale-up often elaborated on or emphasised particular activities and structural considerations. These included the use of scalability assessments, distinguishing between different types of scale-up (e.g., vertical versus horizontal), and accounting for multiple organisational levels involved in scaling efforts (e.g., adopting units and scale-up entities). As a result, outcome measures and corresponding data systems (Pillar 1) were required to span both individual implementing units and system-wide levels. These documents also highlighted the broader implications of scale-up for personnel, underscoring the need for expanded interest holder engagement and enhanced team capacity (Pillars 5 and 6).

Similarly, guidance focused on sustainment placed greater emphasis on actions to assess and build infrastructure that could be supported over the long term (all Pillars). This included evaluating the availability of systems (data, training, communication etc.) and supports that could be maintained with existing resources of target organisations or settings. In particular, sustainment guidance emphasised the importance of robust data collection systems capable of ongoing monitoring (Pillar 1), highlighting that one-off assessments are typically insufficient for measuring sustainability outcomes.

Across both scale-up and sustainment guidance, working within and across resource-constrained settings was a common consideration. These documents frequently recommended strategies to optimise existing infrastructure, leverage partnerships, and build capacity using available resources. This reinforces the broader principle—reflected throughout our synthesis and elaborated in the Discussion section—that effective improvement activities are not solely about following prescribed steps but requires tailoring actions to context, including the realities of resource availability and system readiness.

## Discussion

This systematic review draws together LHS approaches to support the implementation, scale-up, and sustainment of evidence-informed health policies and interventions. We developed a consolidated guidance comprising six pillars that provide the infrastructure, capacity, and expertise to support ongoing improvement activities. It also identified ten sequential yet flexible steps, organised across three LHS phases covering the continuous improvement process. This work aims to strengthen health systems by supporting their generation and application of implementation (inclusive of scale-up and sustainment) research to more rapidly improve the safety and quality of healthcare, patient outcomes and community health.

Existing LHS frameworks focus on descriptions of the core infrastructure, or pillars needed to support improvement and ongoing learning [13–17]. For example, Reid et al [14] conceptualised an LHS as an engine with learning gears (such as implementation and evaluation) and factors such as leadership and funding providing the “fuel” needed for the gears to produce equity-centred outcomes. They also identified several “moderators” and “brakes” that may impede such outcomes. Similarly, Menear et al [15] proposed a conceptual framework for value-creating LHSs that integrates four main dimensions – core values, pillars, processes, and outcomes. Their framework highlights seven pillars that support LHS functionality: scientific, social, technological, policy, legal, ethical, and socio-political infrastructure. These pillars are underpinned by core values such as transparency, equity, and collaboration, and are critical to enabling the learning processes that connect data to knowledge and action. Such ‘pillars’ provide an environment conducive to emergent learning, feedback and improvement within health systems. We saw value in consolidating such guidance and found remarkable consistencies in the attributes and functions of pillars across a diverse range of LHSs, and guidance documents. Such findings are similar to other recent reviews of LHS frameworks [13].

LHS frameworks provide less guidance on the process of improvement—that is, the specific steps needed to enhance healthcare. We addressed this by synthesising the steps recommended by process models of three important and distinct outcomes that help maximise the impact of evidence-based healthcare: implementation, scale-up, and sustainment. It is often suggested that each of these outcomes are considered across each phase of an improvement process. For example, sustainment should be considered when selecting an intervention to implement, and in the process of implementation. We are not aware, however, of guidance that has attempted to bring these together. While the application of the process may differ, such as the specific barriers, strategies employed to address them, or measures used to assess outcomes, we found little differences in the broad steps or process reported in documents reviewed between those with an emphasis on implementation, scale-up or sustainment. This finding is not to suggest that determinants of success across these stages are identical, but rather that frameworks tend to articulate common process logic at a high level. It is also important to note that relatively few included guidance documents explicitly focused on scale up and sustainment. Future research is needed to empirically test the steps and pillars in practice, and to explore their relevance and nuance of application for different purposes (e.g., scale up, sustainment) and across diverse contexts. Regardless, we believe this work is a useful contribution, offering a structured, consistent approach to improvement. Importantly, the process is not intended to be static. In alignment with the continuous nature of LHSs, it can be re-applied over time to support different stages of improvement – from initial implementation through to scale up or sustainment. In this way, it offers both common process and practical flexibility for strengthening evidence-informed practice in dynamic, real-world settings.

Consistent with its intent to consolidate existing implementation improvement frameworks, the phased structure identified in this synthesis aligns with several well-established models, including the Exploration, Preparation, Implementation, Sustainment (EPIS) framework [59]. Our findings also align with adjacent literature on evidence use. For example, our pillars map closely onto the six domains described by Kuchenmüller et al [137] for embedding evidence use in policy: governance, standards/processes, partnership, collective action/support, leadership, resources, and culture. However, while their framework primarily conceptualises these domains as structural and cultural mechanisms that confer legitimacy and sustain evidence use, our synthesis focuses on their practical operationalisation within LHSs, specifically in relation to implementation, scale-up, and sustainment of health interventions. In particular, our findings place emphasis on the systems and processes needed to generate new practice-based evidence, with dedicated Pillars for data systems and evidence synthesis. Such elements are not foregrounded in Kuchenmüller et al. [137], which assumes the availability of existing evidence for use. This focus on evidence generation, as well as use, underscores the dual role of LHSs as both users and producers of evidence. In this respect, our findings also relate to Normalization Process Theory, which conceptualises the embedding of complex interventions as an ongoing process of collective work required to integrate new practices into routine settings [138]. Additionally, while Kuchenmüller et al. [137] frame institutionalisation as a process unfolding in stages, our findings describe a cyclical, practice-oriented improvement process, comprising actionable steps. These two perspectives—the institutionalisation of evidence use and the operationalisation of implementation improvement cycles—offer complementary pathways toward strengthening LHSs. Future research could explore how these perspectives can be integrated to support durable, evidence-informed health systems globally.

Synthesising Steps was complex process due to variation in sequencing across documents. Despite this, we generated a coherent and comprehensive set of steps through iterative coding processes, prioritising placement aligned with practical needs of policy-makers and targeted end users. To enhance usability and relevance across settings, we deliberately kept the steps broad. In some cases, this meant including several key actions (i.e., sub-steps) within a single step, to better reflect the fluid and often non-linear nature of real-world improvement processes.

The Phase 1 steps were especially challenging to position. For example, the identification and establishment of an operational team appeared at various points across guidance documents. In some cases, it was presented more as a pillar. For example, the Competitive Intelligence Process described a network of people (including executives and those with designated positions) as an infrastructure to operationalise the process, but not a step in the process itself [53]. Another challenge was interest holder identification and engagement – this was a recurring activity across all phases of the guidance. We addressed this aspect by including it within Step 1.2 as well as within one of the six pillars, recognising its importance across the improvement process. We also included the key interest holder-related information and activities within respective steps – for example, engaging interest holders to help identify and understand the problem (Step 1.1), and formally identifying interest holders needed for improvement activities as part of planning in Step 1.2. Despite challenges, the overarching LHS phases [24] provided a useful structure for organising the steps, and offered a strong scaffold for coding and situating the diverse improvement activities described across the guidance documents.

A number of methodological strengths of the review are worth noting. The coding process was primarily inductive, allowing findings to emerge directly from the source data, supported by strong LHS-based codebooks and a collaborative coding team. Where gaps or inconsistencies arose, deductive searches and peer discussions clarified concepts and validated interpretations. This highlights the value in having content expertise within the coding team and involved in synthesis, to identify potential gaps and ensure thorough coding. All coded data were reviewed by at least three members of the research team: original coders, secondary reviewers, and a third reviewer during the final data cleaning process ensuring consistency and rigor.

Unlike traditional reviews, we did not appraise the quality of primary studies. This decision reflects the research purpose (to produce an implementation-focused LHS framework) and the nature of the evidence synthesised, which comprised guidance describing improvement logic and operational steps, rather than empirical studies reporting discrete findings. Instead, we prioritised inclusion and transparency, reviewing each document in full and extracting all content based on interpretive judgement of what was relevant, coherent, and adequately described—key considerations for rigorous qualitative evidence syntheses [139]. This approach facilitated the contribution of key content across the breadth of included documents, regardless of whether a document provided a comprehensive model or focused on a single component (e.g., strategy development or evaluation). While some documents contributed more coded content than others, this did not influence the results. Confidence in the final steps and pillars is supported by additional methodological strengths, including an iterative, team-based synthesis process, expertise within the research team, and triangulation across a large dataset [140]. This approach was especially valuable because, although considerable quality assurance procedures were used, including secondary review and targeted re-examination of coded content, it is possible that some relevant material was not captured. This is most pertinent for steps that emerged as distinct later in the coding process, or where activities were mentioned briefly, embedded within other steps, or described implicitly. For example, ‘Monitor and respond’ demonstrated relatively low coding representation (14%) despite ‘audit and feedback’ being one of the most prevalent implementation strategies [141]. Importantly, our synthesis process enabled its identification as a distinct and conceptually important step in the cycle.

The comprehensive nature of steps and pillars also presents a potential limitation. The steps and pillars may appear complex or even infeasible, particularly for settings with limited resources or capacity. Yet this reflects its purpose: to present a thorough version of what LHSs focused on implementation, scale-up, and sustainment can entail. Importantly, the review findings do not assume that all components must be enacted in full, and while the ‘steps’ are presented linearly, in complex adaptive systems like healthcare, we acknowledge this will rarely be the case. Many steps are likely to be iterative, for example, the action plan from Step 1.2 is likely to be amended based on identified gaps during capacity assessments (Step 1.3) or emergent challenges (Step 2.3). The application of findings from this review will also be different by setting and context; the steps and pillars should be interpreted and tailored to the context in which they are applied. This tension between ideal and feasible is not unique to our synthesis—most individual guidance documents included in this review explicitly acknowledged the challenges posed by contextual constraints and resourcing limitations. For example, the IDEAS (Integrate, Design, Assess, and Share) framework drew attention to the variability of its application based on resource constraints [142].

Nonetheless, there are likely to be challenges in operationalising comprehensive LHSs consistent with the steps and pillars describe in this review [143]. Health systems operate within complex, resource-constrained environments, and establishing and sustaining multi-disciplinary teams, building the necessary infrastructure, and embedding continuous learning cycles into routine practice can be resource- and time-intensive. Cost and efficiency are central concerns for LHSs [125, 144], and future research should examine the resource implications and efficiency gains associated with operationalising an implementation-focused LHS, including ways to reduce costs and increase efficiencies. It may also require shifts in culture, processes, and roles. Many guidance documents acknowledged the importance of a supportive organisational culture in enabling reorientation for improvement efforts. For example, the LHS Architectural Framework emphasised the need for internal alignment and a culture of inquiry to embed continuous learning into routine operations [145]. For academics and public health researchers, it also represents a new way of working [24], requiring closer, ongoing partnerships and a willingness to adapt timelines and traditional processes. Potential risks include insufficient leadership buy-in, fragmented data systems, or a lack of resourcing to enact the full cycle.

## Conclusion

The potential for health systems to improve the quality and impact of healthcare is unrealised. Developing LHSs is challenging, even in high income countries and well-resourced healthcare organisations. This review sought to facilitate increased use of LHSs by consolidating diverse guidance, to enhance the generation and application of research to support better implementation, scale-up and sustainment of effective health interventions. Here we provide key findings of our synthesis. The WHO will soon publish a practical guide to help operationalise implementation-focused LHSs. Further efforts should focus on testing and refining the framework in real world contexts to further strengthen its relevance and impact.

## Supplementary Information

Supplementary Material 1. Search strategy

Supplementary Material 2. Website searches

Supplementary Material 3. Characteristics of included documents

Supplementary Material 4. Pillars coding per document

Supplementary Material 5. Steps coding per document

Supplementary Material 6. Pillars quantitative comparison

Supplementary Material 7. Steps quantitative comparison

## Data Availability

Data is available to be shared upon reasonable request.

## Abbreviations

LHS: Learning Health System
WHO: World Health Organization
EVIPNet: Evidence-informed Policy Network
PRISMA: Preferred Reporting Items for Systematic Reviews and Meta-Analyses
AIM: Adapted Intervention Mapping
IDEAS: Integrate, Design, Assess and Share
