# Supplementary material for: Learning health system for implementation, scale-up, and sustainment: a systematic review to consolidate guidance for improvement
Source: Implement Sci. 2026 Jan 10;21:13. doi: 10.1186/s13012-025-01482-z (PMC12910773; doi:10.1186/s13012-025-01482-z)
Supplement: Supplementary file 1 — Supplementary Material 1. Search strategy [file 13012_2025_1482_MOESM1_ESM.docx]

**Search strategy**

Medline (OVID) search date 12^th^ April 2024

| **#** | **Searches** |  |
| --- | --- | --- |
|  | implement*.mp. | 751071 |
|  | adopt*.mp. | 377696 |
|  | (system* adj2 change*).mp. | 21380 |
|  | quality improvement*.mp. | 75409 |
|  | sustain*.mp. | 504778 |
|  | institutionali*.mp. | 23038 |
|  | routin*.mp. | 503992 |
|  | scale*.mp. | 1237543 |
|  | “learning health* system*” | 1341 |
|  | (guideline* OR guidance OR (practice ADJ2 (guide*1 OR recommend* OR standard*)) OR (decision* ADJ2 (making OR make*)) OR (evidence-based ADJ2 (practice* OR medicine OR nursing))).ti,ab. | 925280 |
|  | (or/1-9) adj5 10 | 41619 |
|  | (Health care or healthcare).ti,ab. | 769065 |
|  | Clinical practice.ti, ab. | 265414 |
|  | Health service*.ti,ab. | 140773 |
|  | Public health.ti,ab. | 345189 |
|  | Community health.ti,ab. | 31158 |
|  | Community service.ti,ab. | 1995 |
|  | Medical Care.ti,ab. | 62880 |
|  | Health polic*.ti,ab. | 36589 |
|  | or/12-19 | 1462241 |
|  | 11 and 20 | 15018 |
|  | Randomized Controlled Trial/ | 610363 |
|  | clinical trial/ or controlled clinical trial/ | 564058 |
|  | random allocation/ | 107073 |
|  | Double-Blind Method/ | 177958 |
|  | Single-Blind Method/ | 33354 |
|  | placebos/ | 35934 |
|  | Research Design/ | 1504529 |
|  | Evaluation Studies/ | 127580 |
|  | Comparative Study/ | 262079 |
|  | exp Longitudinal Studies/ | 1913805 |
|  | exp Longitudinal Studies/ | 170646 |
|  | exp Cohort studies/ | 56433 |
|  | Controlled Before-After Studies/ | 2590910 |
|  | Interrupted Time Series Analysis/ | 752 |
|  | comparative study.pt. | 2019 |
|  | clinical trial.tw. | 1913805 |
|  | latin square.tw. | 201638 |
|  | (time adj series).tw. | 5549 |
|  | (before adj2 after adj3 (stud* or trial* or design*)).tw. | 48137 |
|  | ((singl* or doubl* or trebl* or tripl*) adj5 (blind* or mark)).tw. | 17351 |
|  | placebo*.tw. | 202356 |
|  | random*.tw. | 255103 |
|  | (matched adj (communit* or school* or population*)).tw. | 3335 |
|  | control*.tw. | 4784578 |
|  | (comparison group* or control group*).tw. | 616421 |
|  | matched pairs.tw. | 8124 |
|  | outcome stud*.tw. | 9437 |
|  | (quasiexperimental or quasi experimental or pseudo experimental).tw. | 22258 |
|  | (nonrandomi?ed or non randomi?ed or psuedo randomi?ed or quasi randomi?ed).tw. | 40636 |
|  | prospectiv*.tw. | 911314 |
|  | volunteer*.tw. | 221662 |
|  | (or/22-52) | 9683503 |
|  | 21 not 53 | 9912 |
|  | limit 54 to (last 10 years) | 6853 |
