# Supplementary material for: Learning health system for implementation, scale-up, and sustainment: a systematic review to consolidate guidance for improvement
Source: Implement Sci. 2026 Jan 10;21:13. doi: 10.1186/s13012-025-01482-z (PMC12910773; doi:10.1186/s13012-025-01482-z)
Supplement: Supplementary file 2 — Supplementary Material 2. Website searches [file 13012_2025_1482_MOESM2_ESM.docx]

# Supplementary website searches

<https://www.nccmt.ca/organizational-change>

<https://www.ahrq.gov/>

<https://thecenterforimplementation.com/toolbox>

<https://impsciuw.org/>

<https://aho.afro.who.int/af>

<https://campus.paho.org/en>

<https://www.paho.org/en/technical-and-scientific-products>

<https://www.who.int/southeastasia/activities>

<https://www.emro.who.int/e-library/index.html>

<https://www.who.int/westernpacific/our-work/resources>

<https://www.who.int/europe/about-us/our-work>

<https://evidence-impact.org/>

<https://www.emro.who.int/evidence-data-to-policy/training-package/index.html>
